# Mechanistic insights of magnolol antimicrobial activity against *Mycoplasma* using untargeted metabolomic analyses

**DOI:** 10.3389/fcimb.2023.1325347

**Published:** 2023-12-12

**Authors:** Hu Qiao, Zhang Tengfei, Zhang Wenting, Lu Qin, Guo Yunqing, Cao Xiaoyi, Shao Huabin, Zhai Xinguo, Luo Qingping

**Affiliations:** ^1^ Key Laboratory of Prevention and Control Agents for Animal Bacteriosis (Ministry of Agriculture and Rural Affairs), Hubei Provincial Key Laboratory of Animal Pathogenic Microbiology, Institute of Animal Husbandry and Veterinary, Hubei Academy of Agricultural Sciences, Wuhan, China; ^2^ College of Life Science and Food Engineering, Hebei University of Technology, Hebei, China; ^3^ Hubei Hongshan Laboratory, Wuhan, China

**Keywords:** *Mycoplasma synoviae*, magnolol, growth, biofilm, pathogenicity, cell membrane, metabolomics, lipid

## Abstract

The unreasonable use of antibiotics is one of the important causes of antimicrobial resistance (AMR) that poses a huge public health threat. Magnolol is a traditional Chinese medicine exhibiting antibacterial-, antifungal-, anti-inflammatory-, and antioxidant activities. However, it is unclear whether magnolol has an inhibitory effect on *mycoplasma*. This study found that magnolol showed excellent inhibitory activity against various mycoplasmas. Magnolol showed dose-dependent inhibition of *Mycoplasma synoviae* growth and biofilm formation *in vitro*. Magnolol caused severely sunken and wrinkled *M. synoviae* cell membranes at the minimum inhibitory concentration, and an enlarged cell diameter. The chicken embryo infection model showed that magnolol significantly reduced *M. synoviae* pathogenicity *in vivo*. Kyoto Encyclopedia of Genes and Genomes pathway analysis showed that the citrate cycle, glycolysis/gluconeogenesis, and pyruvate metabolism were significantly disturbed at the minimum inhibitory concentration of magnolol. Interestingly, 41% of differential metabolites were in the categories of lipids and lipid-like molecules. Protegenin A was up-regulated 58752-fold after magnolol treatment. It belongs to fatty acyls, and destroys cell membrane integrity and cell activity. Ghosphatidylethanolamine, phosphatidylglycerol, phosphatidic acid, and phosphatidylserine related to membrane maintenance and stress response were widely down-regulated. Collectively, our results illustrate the feasibility of magnolol as a phytochemical compound to treat mycoplasma infection.

## 1 Introduction

*Mycoplasma* belongs to the Firmicute phylum, Mollicute class, *Mycoplasmatales* order, and *Mycoplasmataceae* family, which are mainly characterized by the smallest self-replicating microorganism lacking a cell wall (Rebollo Couto et al., 2012). *Mycoplasma* has a high degree of host and tissue specificity and mainly colonizes epithelial surfaces of the genitourinary tract and respiratory, mammary gland, and serosa. Most *mycoplasma* such as *Mycoplasma hyopneumoniae*, *Mycoplasma gallisepticum* and *Mycoplasma synoviae* are pathogens that cause serious diseases and great economic losses in livestock production (Lloyd et al., 1986).

*M. synoviae* and *M. gallisepticum* are important pathogens in poultry industry that are the major causes of economic losses to the poultry industry (Feberwee et al., 2022). Recently, chronic respiratory disease (CRD), infectious synovitis, and eggshell apex abnormalities caused by *M. synoviae* have led to serious damage to poultry industry (Kursa et al., 2019; Petrone-Garcia et al., 2020). Therefore, *M. synoviae* has attracted extensive attention as a serious pathogen. Attenuated live vaccine of MS-H (Vaxsafe^®^MS) is used to prevent *M. synoviae* wildtype strain infection in poultry (Shahid et al., 2014). In addition, antibiotics including macrolides, lincosamides, pleuromutilins, and fluoroquinolones are another effective way to prevent and treat *M. synoviae* infection in veterinary clinics (Petrone-Garcia et al., 2020). However, the antimicrobial resistance (AMR) of *mycoplasma* is becoming serious owing to the unreasonable use of antibiotics in veterinary clinical treatment (Davies and Davies, 2010). Recently, *M. synoviae* resistance to enrofloxacin has become widespread in China (Zhang et al., 2022). If it doesn’t attract enough attention, AMR will become a huge threat to public health (Samreen et al., 2021). Hence, the development of novel antimicrobial agents is urgently needed.

Magnolol is a traditional Chinese medicine derived from *Magnolia officinalis* Cortex that displays potent antibacterial-, antifungal-, anti-inflammatory-, and antioxidant activities (Zhang et al., 2019). Magnolol or its derivatives have remarkable antibacterial and antifungal activities against *Candida albicans* (Behbehani et al., 2017; Xie et al., 2022), *Streptococcus mutans* (Greenberg et al., 2007); *Staphylococcus aureus* (Guo et al., 2021), *Alternaria alternata* (Wang et al., 2020), *Rhizoctonia solani* (Mo et al., 2021), *Fusarium* (Oufensou et al., 2019), *Magnaporthe grisea* (Choi et al., 2009), and *Penicillium expansum* (Chen et al., 2019). Magnolol could damage the integrity of fungal cells and interfere with cell metabolism to inhibit the growth of *R. solani* and *A. alternata* (Wang et al., 2020; Mo et al., 2021). Magnolol caused significant inhibition of gene expression that are related to adhesion, invasion, hyphal formation, biofilm formation, and metabolic enzymes in *C. albicans* (Xie et al., 2022). Magnolol or its derivatives have excellent antibacterial and anti-infective activity by destroying bacterial cell membranes (Guo et al., 2021).

Some studies have revealed the mechanism of antifungal and antibacterial activity of magnolol. However, it is unclear whether magnolol can inhibit *mycoplasma* activity. This study found that magnolol inhibited various *mycoplasma*. *M. synoviae* was used as a model to reveal that magnolol destructed cell morphology, biofilm formation, and colonization ability. Metabolomics analysis were conducted to systematically investigate the mechanism of magnolol against *M. synoviae*. In general, this study provided a theoretical basis for the further development of magnolol as a therapeutic agent in combating *mycoplasma* infections.

## 2 Materials and methods

### 2.1 Strains and culture conditions

The strains of *M. synoviae*, *M. gallisepticum*, and *M. hyopneumoniae* were maintained at Hubei Academy of agricultural Sciences, which were cultured in modified Friis’ medium at 37°C with 5% CO_2_ as described previously (Frey et al., 1968; Dusanic et al., 2009; Liu et al., 2022). Macrolides and fluoroquinolone are commonly antibiotics that used to treat *M. synoviae* infection. The MIC of tylosin and ciprofloxacin against *M. synoviae* showed 0.98 µg/mL and 3.91 µg/mL, respectively.

### 2.2 Minimal inhibitory concentration and minimum bactericidal concentration

Magnolol were serially diluted two-fold with concentration ranging between 2000 µg/mL to 0.98 µg/mL in 96-well plates, then mixed with 100 μL of *mycoplasma* culture (1 × 10^6^ CCU/mL) to a final concentration of 1000 µg/mL to 0.49 µg/mL. Plates were incubated at 37°C with a 5% CO_2_ humidified incubator for 48 h. The color of media in each well was monitored. The MIC was determined as the minimal concentration of magnolol that resulted in no color change. The assay was performed in triplicate.

The MBC was determined by plating the culture in wells with growth inhibition (no color change) onto modified Friis’ solid medium, and incubated at 37°C with 5% CO_2_ humidified incubator for at least 7 d. The lowest magnolol concentration that resulted in no *mycoplasma* growth on solid medium was considered the MBC. The assay was performed in triplicate.

### 2.3 Time-kill kinetic assay

A time-kill kinetics assay was conducted to evaluate the bactericidal performance of magnolol in killing *M. synoviae*. Briefly, *M. synoviae* cells were grown to the mid-log phase in modified Friis’ medium, then subcultured 1:10 into the corresponding medium supplemented with different concentrations of magnolol (0 ×, ½ ×, 1 ×, 2 ×, and 4 × MIC). Subsequently, the treated suspensions were collected at different time points (12, 24, 36, and 48 h), and the effect of magnolol on growth was recorded by the color-changing units (CCU). The assay was performed in triplicate.

### 2.4 Morphological analysis by scanning electron microscope

Scanning electron microscopy (SEM) was performed as previously described with some modifications (Hu et al., 2021). Briefly, *M. synoviae* culture in the mid-log phase were co-incubated with or without 1×MIC of magnolol at 37°C for 12 h, fixed with 2.5% glutaraldehyde overnight at 4°C, dehydrated with a serial dilution of ethanol, air-dried, covered with a 10 nm gold/platinum layer, and observed by SEM (JFC-1600, JEOL, Japan).

### 2.5 Biofilm formation assay

The effect of magnolol on biofilm formation in *M. synoviae* was assessed by crystal violet staining using a previously described method with some modifications (Chen et al., 2012). Briefly, *M. synoviae* was grown in modified Friis’ medium at 37°C in a 5% CO_2_ humidified incubator for 36 h. The culture was then subcultured 1:10 into the corresponding medium with or without magnolol in a 96-well flat-bottom microplate, and incubated for 72 h. The wells were gently washed three times with PBS, stained with 1% crystal violet for 10 min at room temperature, washed four times with distilled water, and air dried. The dye was released by adding 100 μL of 33% acetic acid and quantified by recording the absorbance at 595 nm.

To evaluate that magnolol eliminates the mature biofilms of *M. synoviae* at the different concentration. The method described as above with some modifications. Briefly, the culture was incubated for 72 h to form the mature biofilm in a 96-well flat-bottom microplate, then added magnolol to a final concentration of 0, ¼ MIC, ½ MIC, 1 MIC, and re-incubated for 24 h, stained with crystal violet, measured the absorbance at 595 nm.

### 2.6 Biofilm visualization by confocal microscopy

Confocal microscopy was used to analyze the biofilm of *M. synoviae* as previously described with some modifications (Yi et al., 2020). Briefly, biofilms were formed with or without magnolol on round coverslips in a 12-well plate. After a 72-h incubation, the coverslips were gently washed three times with PBS to remove poorly attached cells and stained with LIVE/DEAD^®^ BacLight™ Bacterial Viability and Counting Kit according to the manufacturer’s protocol (ABI L34856; Invitrogen, USA). SYTO 9 was used to label the live bacteria, which fluoresced green (488 nm). Propidium iodide was used to label the dead bacteria, which fluoresced red (561 nm). The sample was subsequently incubated at room temperature for 10 min, washed three times with PBS, and imaged by confocal microscopy.

### 2.7 Protection of chicken embryo by magnolol against *M. synoviae* infection

Previous study has reported that the magnolol exerted a low cytotoxicity in broilers, which can improve growth performance by modulating mucosal gene expression and the gut microbiota in the treatment of 300 mg/kg (Chen et al., 2021). Chicken embryos were used as an infection model to evaluate the protection provided by magnolol as previously described with some modifications (Helmy et al., 2020; Zhang et al., 2020). Briefly, Specific pathogen-free (SPF) chicken embryos were purchased from Merial-Vital, Beijing, China, and hatched in an environment of 37.5°C and 50–60% humidity. Five-day-old chicken embryos with similar body weights were randomly divided into 6 per group, challenged with 5 x 10^8^ CCU of *M. synoviae* via allantoic cavity, followed by injection with magnolol at 1 mg/kg•body weight after 2 d post infection; PBS was used as a negative control. Chicken embryos were euthanized after 10 d post infection. The upper part of the trachea and right side of the lung were collected, weighed, homogenized in sterile saline, plated onto modified Friis’ solid plates, and counted with a light microscope after 7 d of incubation.

### 2.8 Non-targeted metabolomic analysis

*M. synoviae* cells were cultured in modified Friis’ medium to mid-log phase, then co-incubated with 1× MIC of magnolol at 37°C for 12 h. The untreated group was used as the control. The cells were harvested at 3,000 rpm, resuspended with methanol/chloroform, ultrasonicated, then added L-2-chlorophenylalanine (0.3 mg/mL) dissolved in methanol as internal standard, and ultrasonicated again. The supernatants were dried in a freeze concentration centrifugal dryer, redissolved in a mixture of methanol and water, vortexed for 30 s, ultrasonicated, then treated for 2 h at -20°C. The supernatants were collected using crystal syringes, filtered through 0.22 μm microfilters, and transferred to LC vials. ACQUITY UPLC I-Class system (Waters Corporation, Milford, USA) coupled with VION IMS QTOF Mass spectrometer (Waters Corporation, Milford, USA) was used to analyze the metabolic profiling in ESI positive and ESI negative ion modes. An ACQUITY UPLC BEH C18 column (1.7 μm, 2.1 × 100 mm) was used in positive and negative modes. The software of AbfConverter.4.0.0, MS-DIAL, and Progenesis Progenesis QI were used for Mass Spectrometry data conversion and Analysis. The software of RStudio and Notepad++ were used to data processing, calculation and drawing.

### 2.9 Statistical analysis

GraphPad 8 was used for graph creation. ImageJ was used to measure cell length and width. One-way analysis of variance (ANOVA) and Tukey’s test (α= 0.05) were used to determine significant differences (https://astatsa.com/OneWay_Anova_with_TukeyHSD/ ).

## 3 Results

### 3.1 Activity of magnolol against *Mycoplasma*

**Figure 1:**
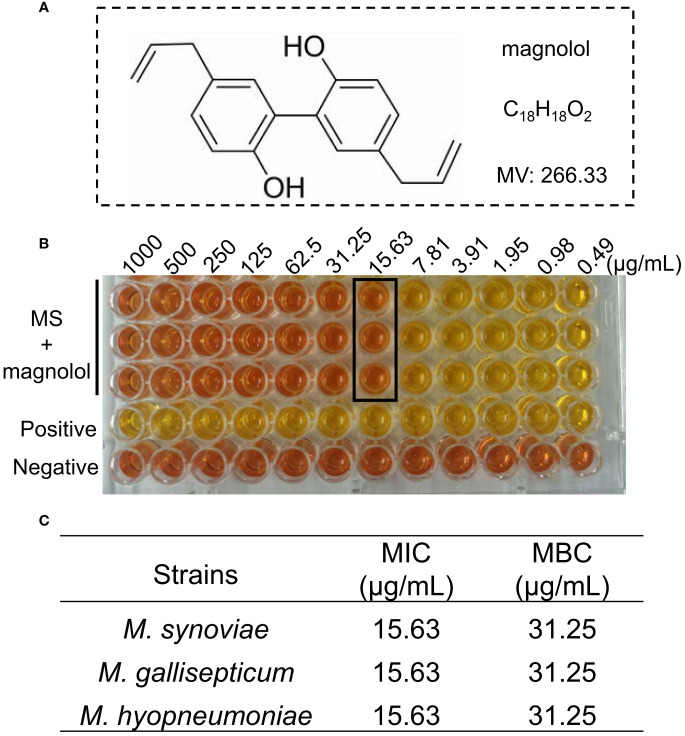
The activity of magnolol against *Mycoplasma*. **(A)** Chemical structure of magnolol. **(B)** Minimal inhibitory concentration (MIC) of magnolol against *M. synoviae*. **(C)** MIC and minimum bactericidal concentration (MBC) values of magnolol against *M. synoviae*, *M. gallisepticum*, and *M. hyopneumoniae*. Data are presented as means ± SD of triplicate assays.

Magnolol had activity against *M. synoviae*, with a MIC of 15.63 µg/mL and MBC of 31.25 µg/mL. It also exhibited inhibitory effects against other *mycoplasma* strains, such as *M. gallisepticum* and *M. hyopneumoniae* (**Figures 1B, C**). Above all, this suggested that magnolol is a potential antibacterial agent *Mycoplasma*.

### 3.2 Killing effect of magnolol against *M. synoviae*

**Figure 2:**
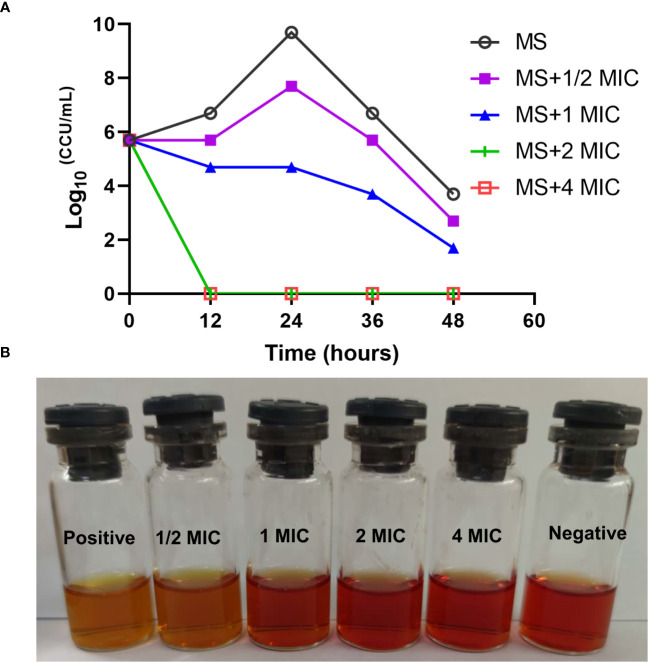
Time-kill kinetics of magnolol against *M. synoviae*. **(A)** Killing effect of magnolol against *M. synoviae*. The magnolol concentrations ranged from ½ × MIC to 4 × MIC. The viability of *M. synoviae* was monitored by a color changing unit (CCU) assay at the indicated times. **(B)** Visualization of the effect of magnolol on *M. synoviae* growth. The culture medium was photographed after 48 h of incubation.

*M. synoviae* cell growth was inhibited at a MIC of 15.63 µg/mL of magnolol compared to untreated cells and were completely eradicated within 12 h under 2 × MIC of magnolol (31.25 µg/mL) (**Figure 2**). The bactericidal performance of magnolol was dose-dependent.

### 3.3 Destructive effect of magnolol on *M. synoviae* structure

**Figure 3:**
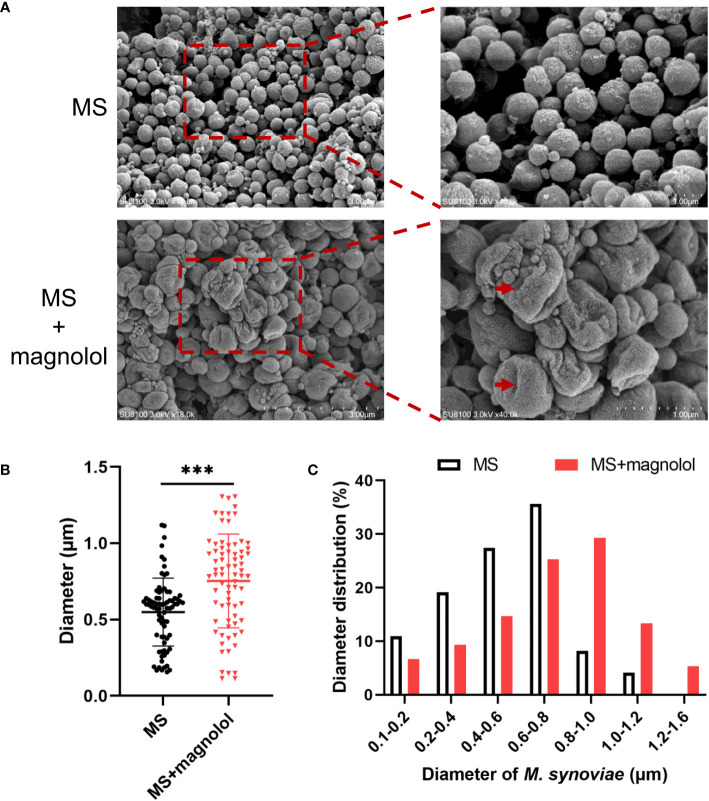
Morphological analyses of *M. synoviae*. **(A)** Cell morphology was analyzed with SEM in the treatment of magnolol at the MIC. The scale bars are 3 µm and 1 µm. The red arrow indicated cellular damage caused by magnolol. **(B)** *M. synoviae* diameter. Data are presented as the mean ± SD of triplicate assays. ***p value <0.001. **(C)** Histogram statistics of cell diameter.

The magnolol treated cells showed severely sunken and wrinkled of cell membranes (marked by red arrows) compared with untreated cells that displayed a standard spherical shape (**Figure 3A**). Cell diameter was measured using ImageJ as previously described (Vischer et al., 2015). On average, the cell diameter treated with magnolol was significantly larger (0.75 ± 0.31 µm) compared with the untreated cells (0.55 ± 0.22 µm) (**Figure 3B**). Moreover, the distribution of distinct cell diameter classes differed between the untreated and treated cells. The untreated cells were concentrated in a cell diameter interval of 0.1 to 1.2 µm (n= 73), and approximately 35.6% (26/73) of that showed a diameter of 0.6 to 0.8 µm that represented the highest proportion. However, the diameters of treated cell were distributed between 0.1 to 1.6 µm (n= 75), and 29.3% (22/75) showed a diameter of 0.8 to 1.0 µm, which represented the highest proportion (**Figure 3C**). Overall, the results suggested that magnolol severely damaged the cell membrane and cell morphology of *M. synoviae*.

### 3.4 Magnolol decreased *M. synoviae* biofilm formation

**Figure 4:**
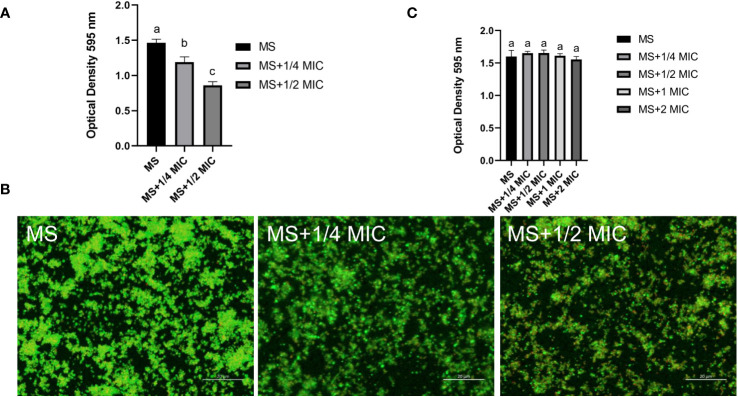
Biofilm formation of *M. synoviae* cultured at sub-MICs of magnolol. **(A)** Crystal violet staining of biofilm in the wells of a 96-well microplate. The absorbance was read at 595 nm. **(B)** Confocal microscopy showing the *M. synoviae* biofilm structures labeled with fluorescent SYTO 9 (green fluorescence) and propidium iodide (red fluorescence). **(C)** Crystal violet staining of mature biofilm after treated with magnolol. The absorbance was read at 595 nm. Data are presented as the mean ± standard deviation of triplicate assays. Different letters indicate significant differences according to Tukey’s test (α = 0.01).

Sub-MICs levels (½ and ¼ MIC) of magnolol were used to test the influence of magnolol on biofilm formation, Magnolol significantly reduced the formation of *M. synoviae* biofilm (**Figure 4A**). The inhibitory efficacy increased with higher magnolol concentrations. Confocal microscopy combined with SYTO 9 and propidium iodide were used to compare that of treated and untreated cells to visualize the inhibition of magnolol on biofilm formation. Dense bacterial masses appeared in the untreated cells, whereas the magnolol treated cells were more dispersed and less aggregated (**Figure 4B**). Interestingly, biofilm elimination assay found that the mature biofilm was not eliminated on the treatment with ¼ MIC, ½ MIC, 1 MIC, 2 MIC of magnolol (**Figure 4C**). The results showed that magnolol could significantly inhibited the biofilm formation of *M. synoviae* with a dose-dependent, but could not eliminate mature biofilms.

### 3.5 Magnolol protected chicken embryo against *M. synoviae* infection

**Figure 5:**
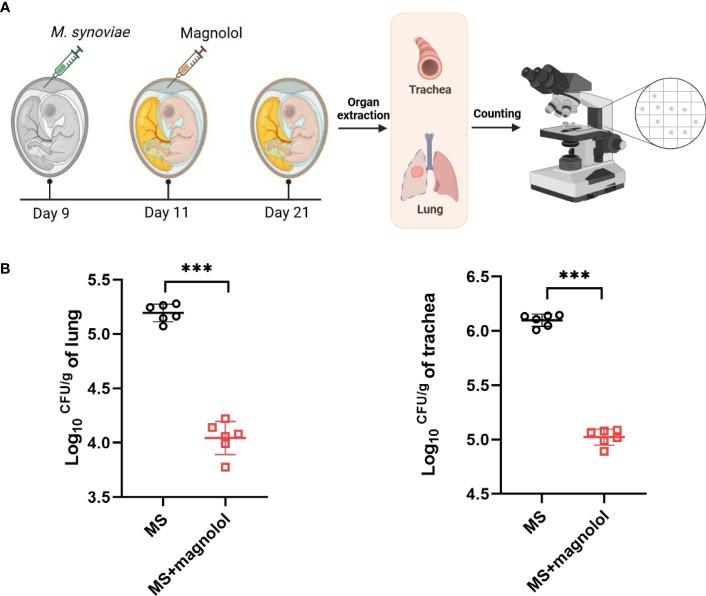
The evaluation efficacy of magnolol against *M. synoviae* in chicken embryos. **(A)** Schematic diagram of chicken embryo infection. **(B)** Colonization assay. Chicken embryos were infected with 5 x 10^8^ CCU of *M. synoviae* via allantoic cavity on day 9, then treated with magnolol at 1 mg/kg•body weight after 2 d post infection. Chicken embryos were euthanized after 10 d post infection. The lung and trachea were collected, resuspended in PBS, homogenized, and plated on modified Friis’ medium agar plates for colony enumeration; PBS was used as a negative control (n=6). Statistical significance was determined by two-tailed, unpaired Student’s *t*-tests. Error bars represented the mean ± standard deviations (ns, *p* value > 0.05; ***p value < 0.001).

We performed the colonization assay using a chicken embryo infection model to evaluate the protective efficacy of magnolol against pathogenic bacteria *in vivo* (**Figure 5A**). The group treated with 1 mg/kg•body weight of magnolol presented a significantly lower bacterial load than that of the untreated group in the lung and trachea (**Figure 5B**). The results showed that magnolol had potent antimicrobial activity *in vivo*.

### 3.6 Metabolomic analysis of *M. synoviae* in treatment with magnolol

**Figure 6:**
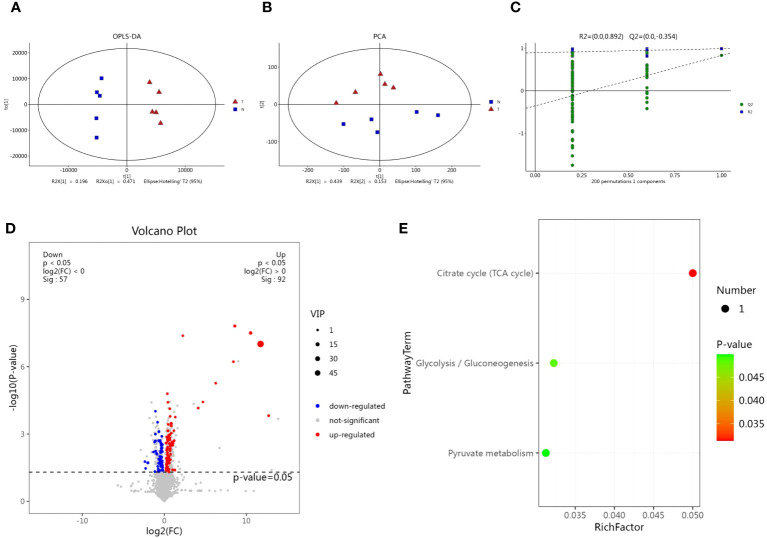
Metabolomic analysis of *M. synoviae* following treatment with magnolol. **(A)** Orthogonal Projections to Latent Structures Discriminant Analysis (OPLS-DA) score plots of *M. synoviae* after treatment with magnolol. The lines denote 95% confidence interval Hotelling’s ellipses. **(B)** Principle Component Analysis (PCA) scores plot showed obvious separation between the two groups in positive and negative modes. **(C)** The 7-fold cross validation and response permutation testing (RPT) were used to evaluate model validity. **(D)** Volcano plots showing the differential metabolites (DMs) in the magnolol treated group compared to the untreated group. Blue dots represent down-regulated, red dots represent up-regulated, and grey dots represent no significant change (p<0.05, VIP>1, FC>1). N represents the untreated group, T represents the magnolol-treated group. **(E)** Kyoto Encyclopedia of Genes and genomes (KEGG) pathway enrichment of DMs.

We performed a comparative metabolomic analysis with untreated and treated cells using liquid chromatography-mass spectrometry (LC-MS) to investigate the influence of magnolol on the metabolism of *M. synoviae*. The software of SIMCA (version 14.0) was used to analyze the positive and negative data. The Orthogonal Projections to Latent Structures Discriminant Analysis (OPLS-DA) and Principle Component Analysis (PCA) scores plot showed obvious separation between the two groups in positive and negative modes (**Figures 6A, B**). Permutation tests showed that validity of model evaluation (**Figure 6C**). A total of 149 differential metabolites (DMs) were identified, with 92 up-regulated and 57 down-regulated (**Figure 6D**). The KEGG pathway enrichment analysis found that the DMs were mainly clustered in the categories of citrate cycle (TCA cycle), glycolysis/gluconeogenesis, and pyruvate metabolism (**Figure 6E**). The results implied that magnolol widely regulated the metabolism of *M. synoviae*, especially for TCA cycle, glycolysis/gluconeogenesis, and pyruvate metabolism.

### 3.7 Effects of magnolol on lipid metabolic profiling of *M. synoviae*

**Figure 7:**
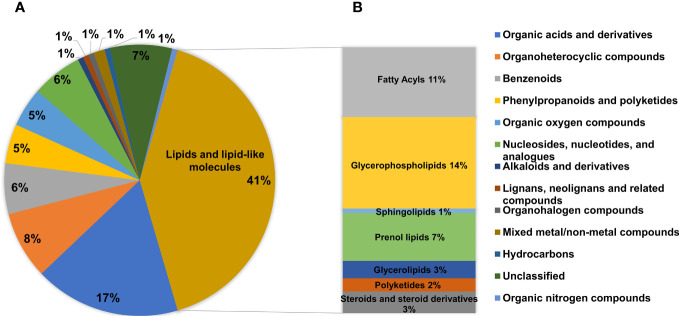
Analysis of the DMs. **(A)** Pie chart showing the proportions of different categories among all DMs. **(B)** The abundance of specific metabolites in the category of lipids and lipid-like molecules.

A total of 41% DMs fell into the category of lipids and lipid-like molecules (**Figure 7A**). This was the dominant category apart from unclassified metabolites. Lipids and lipid-like molecules are essential constituent of cells, formed with a broad range of components such as glycerophospholipids (GPs), fatty acids, sphingolipids (SPs), glycerolipids (GLs), and so on (Fahy et al., 2009). This study showed that the category of lipids and lipid-like molecules were significantly disturbed in the magnolol group, which were consistent with 11% fatty acyls, 14% GPs, 1% SPs, 7% prenol lipids, 3% GLs, 2% polyketides, and 3% steroids and steroid derivatives (**Figure 7B**). The data suggested that GPs and fatty acyls were the most strongly affected metabolites in the category of lipids and lipid-like molecules.

### 3.8 Glycerophospholipids and fatty acyls

**Figure 8:**
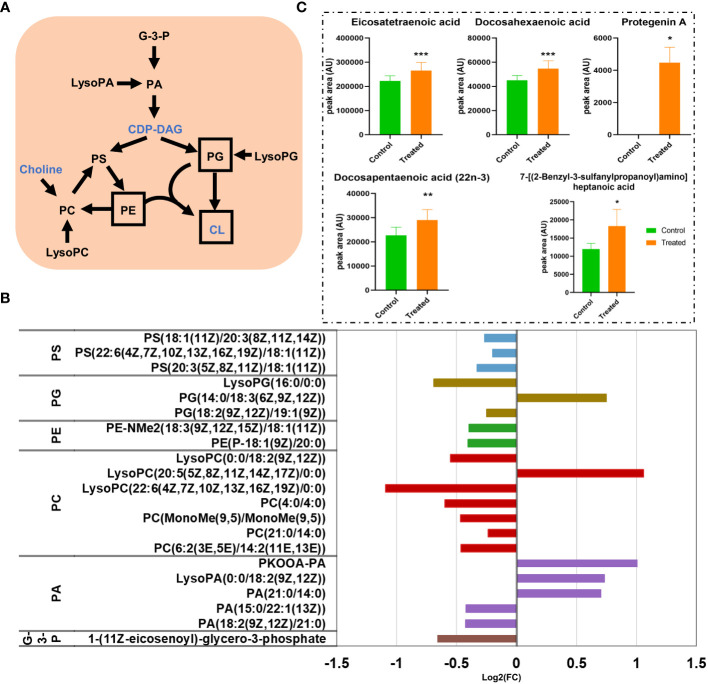
The disturbance of glycerophospholipids (GPs) and the fatty acyl metabolic pathway. **(A)** Scheme of GPs metabolic pathways in *M. synoviae*. The names of the metabolites with altered abundance are shown in black, while no significantly different metabolites are shown in blue. The most important components of the cell membrane are shown in a black frame. **(B)** Alteration of the GP metabolic pathway during treatment with magnolol. G-3-P, 1-glyceraldehyde-3-phosphate; PA, phosphatidic acid; LysoPA, lysophosphatidic acid; CDP-DAG, cytidine diphosphate diacylglycerol; PS, phosphatidylserine; PC, phosphatidylcholine; LysoPC, lysophosphatidylcholine; PE, phosphatidylethanolamine; PG, phosphatidylglycerol; LysoPG, lysophosphatidylglycerol; CL, cardiolipin. **(C)** The alteration of abundance at the sub-class of fatty acids and conjugates during magnolol treatment *p value < 0.05, **p value <0.01, ***p value <0.001.

Glycerophospholipids are major structural components of bacterial membranes that are primarily composed of phosphatidylethanolamine (PE), phosphatidylglycerol (PG), cardiolipin (CL), and so on (Lopez-Lara and Geiger, 2017). The major metabolic pathways of GPs in *M. synoviae* are shown in **Figure 8A**; CDP-diacylglycerol (CDP-DAG) is a precursor for GP synthesis, which is converted by phosphatidic acid (PA), then converted to zwitterionic lipid PE, anionic lipid PG, and CL(Lopez-Lara and Geiger, 2017). This study identified 21 differential metabolites (6 up-regulated and 15 down-regulated) in the pathway of GP synthesis. Every metabolite in the sub-class of phosphatidylcholine (PC), phosphatidylserine (PS), and PE significantly decreased in abundance (**Figure 8B**; **Supplementary Table S1**). The data showed that the metabolic pathway of GPs were significantly affected in the treatment of magnolol.

Fatty acyls are key categories of metabolites that act as a source of energy and form complex lipids (Xicoy et al., 2019). Additionally, it is a component of membranes that extensively regulates biological processes, including intracellular signal-ling, gene expression, transcription factors, inflammation, and bioactive lipid production (Calder, 2015; Fritsche, 2015) This study revealed that fatty acyls are a strongly affected metabolites in the category of lipids and lipid-like molecules. Sixteen DMs in the pathway of fatty acyls were identified, with 10 up-regulated and 6 down-regulated, that are widely distributed in the sub-class of eicosanoids, fatty acids and conjugates, fatty acyl glycosides, fatty alcohols, lineolic acids and derivatives, and oxygenated hydrocarbons (**Supplementary Table S2**). We found that almost every metabolite in the sub-class of fatty acids and conjugates was present in high abundance. It is worth noting that the abundance of “Protegenin A” significantly increased nearly 58,753-fold (**Figure 8C**). The results showed that magnolol significantly affected the metabolic pathway of fatty acyls on *M. synoviae*.

## 4 Discussion

The unreasonable use of antibiotics in veterinary clinic leads to the emergence of AMR that poses a serious threat to human health (Al-Khalaifa et al., 2019). The use of antibiotics in livestock and poultry breeding industry has been greatly reduced with the promulgation of the order to restrict the use of antibiotics, resulting in increasing difficulties in the prevention and control of mycoplasma disease (Kama-Kama et al., 2017; Nhung et al., 2017). Compared to *M. gallisepticum*, *M. synoviae* causing chronic respiratory disease (CRD), infectious synovitis, and eggshell apex abnormalities, which lead to serious damage to poultry industry and attracted extensive attention in recent years (Kursa et al., 2019; Petrone-Garcia et al., 2020). Therefore, there is an urgent need to develop antibiotic substitutes to help livestock and poultry production. This study found that magnolol is a potential agent against *M. synoviae*. Metabolomics was used to analyze the mechanism of magnolol against the growth, cell morphology, biofilm formation, and colonization ability of *M. synoviae*. Moreover, to verify the broad-spectrum properties of magnolol against mycoplasma of other animal origin, *M. hyopneumoniae* was used to evaluated antimicrobial property.

Magnolol is one of major bioactive isolates from *Magnolia officinalis* that has many pharmacological activities, including anticancer-, anti-inflammatory-, antifungal-, and antioxidant activities (Zhang et al., 2019; Xie et al., 2022). This study showed that magnolol significantly inhibited the growth of various mycoplasma *in vitro*, including *M. synoviae*, *M. gallisepticum*, and M. *hyopneumoniae*, The MICs of magnolol against various mycoplasma were determined at 15.63 µg/mL, and the inhibitory effect was dose-dependent (**Figures 1**, **2**). This result is consistent with previous studies demonstrating that the MIC of magnolol against *S. aureus* ATCC29213 was 32 µg/mL, 64 µg/mL for *Enterococcus faecalis* ATCC29212, 16 µg/mL for *Bacillus subtilis* and *Micrococcus luteus*, and below 128 µg/mL for *Escherichia coli* ATCC25922, *Stenotrophomonas maltophilia*, *Salmonella enterica* H9812, and *Salmonella enterica* 8389 (Guo et al., 2021). As for antifungal activity, the MIC of magnolol against *A. alternata* was determined at 100 µM (26.6 µg/mL), EC_50_ of 7.47 µg/mL for *R. solani*, MIC of 40 µg/mL for *C. albicans* (Zhou et al., 2017; Wang et al., 2020; Mo et al., 2021) The results suggested that magnolol exhibited a significant inhibitory effect against mycoplasmas.

**Figure 9:**
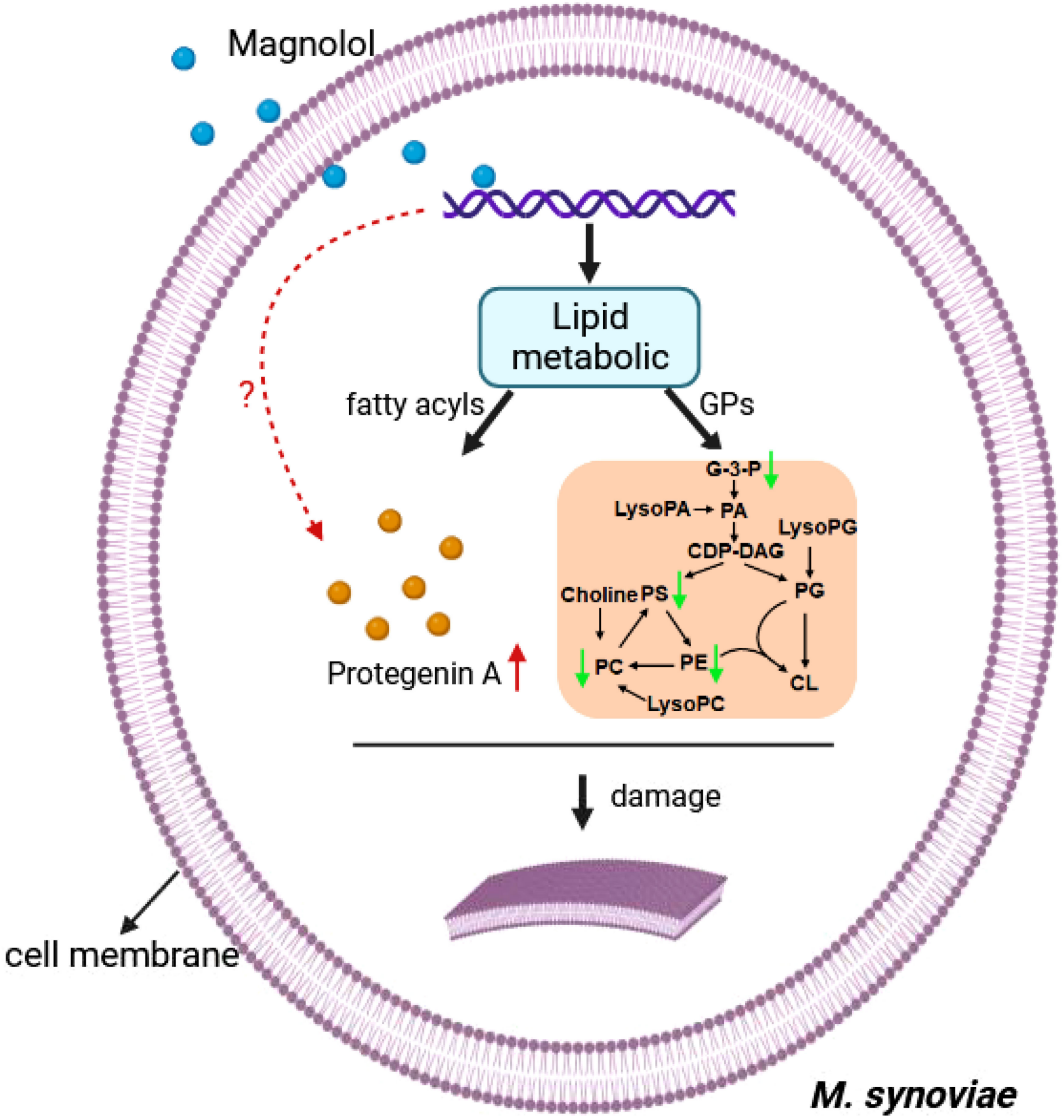
The Schematic diagram demonstrating the membrane damage effect of magnolol against *M. synoviae*. GPs, glycerophospholipids; G-3-P, 1-glyceraldehyde-3-phosphate; PA, phosphatidic acid; LysoPA, lysophosphatidic acid; CDP-DAG, cytidine diphosphate diacylglycerol; PS, phosphatidylserine; PC, phosphatidylcholine; LysoPC, lysophosphatidylcholine; PE, phosphatidylethanolamine; PG, phosphatidylglycerol; LysoPG, lysophosphatidylglycerol; CL, cardiolipin. The green arrow indicates down-regulated, red arrow indicates up-regulated. the red question mark indicates unknown.

Cell morphology analysis showed that magnolol caused severely sunken and wrinkled of cell membranes, and abnormal cell diameter (**Figure 3**), which is consistent with the results reported in previous studies (Behbehani et al., 2017; Wang et al., 2020; Mo et al., 2021). Interestingly, comparative metabolomics analysis revealed that lipid and lipid molecules accounted for the largest proportion of DMs (up to 41%) (**Figure 7A**). Fatty Acyls are modulators of cell membrane properties and a reservoir of energy (de Carvalho and Caramujo, 2018), shows variable production in the group of magnolol treatment. In this study, the abundance of protegenin A increased nearly 58752-fold in the group of treatment. Protegenin (also called bacterial polyynes) act as an antimicrobial agent that is composed of natural compounds containing an ene-tetrayne, ene-triyneene, or ene-triyne (Ross et al., 2014). Polyyne biosynthetic gene clusters are widely distributed within bacteria, such as *Pseudomonas protegens*, *Burkholderia caryophylli*, and *Pseudomonas fluorescens* (Murata et al., 2022). A recent study found that bacterial polyynes disrupt cell membrane integrity and impair the cell viability by inhibiting acetyl-CoA acetyltransferase activity in *C. albicans* (Lin et al., 2022). These findings indicate that magnolol may influence cell membrane integrity and cell viability via disrupting protegenin A production (**Figure 9**). However, the underlying mechanism by which magnolol regulates protegenin A production remains to be further investigated.

Magnolol reduced the biofilm formation and pathogenicity of *M. synoviae*. Biofilm is formed in the bacteria that aggregate in the self-synthesized hydrated polymer matrix (Costerton et al., 1999). Previous studies have shown that biofilm-forming ability is affected by energy-related metabolic pathways, including glycerolipid, amino acid, and carbohydrate metabolism (Lu et al., 2019; Qi et al., 2022). This study found that the inhibitory effect of magnolol on the biofilm formation of *M. synoviae* was enhanced with increasing concentration, which is consistent with previous studies (Behbehani et al., 2017; Guo et al., 2021). Magnolol could not eliminate the mature biofilms of *M. synoviae*. Previous studies have revealed that magnolol significantly decreased the activity of catalase (CAT), polyphenol oxidase (PPO), superoxide dismutase (SOD), succinate dehydrogenase (SDH) and NAD-malate dehydrogenase (NAD-MDH), and so on (Wang et al., 2020; Mo et al., 2021). In our study, KEGG analysis revealed that the pathway of citrate cycle, glycolysis/gluconeogenesis, and pyruvate metabolism were significantly disturbed in the treatment of magnolol (**Figure 6D**). This may potentially explain the effect of magnolol on biofilm formation and pathogenicity. Moreover, the proportion of GPs with changed abundance in class of lipids and lipid-like molecules showed highest, accounting for 14% (**Figure 7B**). PA, PC, PE, PE, PG, and PS belong to GPs and showed widely down-regulated abundance in the magnolol treated group (**Figure 9**). Previous studies revealed that the abundance of lipids is closely related to the formation of biofilms (Lattif et al., 2011; Benamara et al., 2014) The proportions of PE and PG were significantly higher abundance in biofilms bacteria than planktonic bacteria (Benamara et al., 2014). These findings suggest that the energy-related metabolism (especially lipid metabolism) plays an important role in *M. synoviae* biofilm formation.

## Data availability statement

The original contributions presented in the study are included in the article/**Supplementary Material**. Further inquiries can be directed to the corresponding authors.

## Ethics statement

The animal study was approved by Animal experiment was approved by the Institutional Animal Care and Use Committee of the Hubei Academy of Agriculture Sciences, Wuhan, China. The study was conducted in accordance with the local legislation and institutional requirements.

## Author contributions

HQ: Funding acquisition, Investigation, Supervision, Writing – original draft, Writing – review & editing. ZT: Conceptualization, Software, Supervision, Writing – original draft. ZW: Methodology, Software, Writing – original draft. LQ: Formal Analysis, Methodology, Writing – original draft. GY: Resources, Supervision, Writing – original draft. CX: Conceptualization, Methodology, Writing – original draft. SH: Validation, Visualization, Writing – review & editing. ZX: Project administration, Validation, Writing – review & editing. LuoQ: Funding acquisition, Project administration, Visualization, Writing – review & editing.
